# Efficient whole cell biocatalyst for formate-based hydrogen production

**DOI:** 10.1186/s13068-018-1082-3

**Published:** 2018-04-02

**Authors:** Patrick Kottenhahn, Kai Schuchmann, Volker Müller

**Affiliations:** Molecular Microbiology & Bioenergetics, Institute of Molecular Biosciences, Johann Wolfgang Goethe University, Max-von-Laue-Str. 9, 60439 Frankfurt am Main, Germany

**Keywords:** Hydrogen production, Biohydrogen, *Acetobacterium woodii*, Formate dehydrogenase, Hydrogenase

## Abstract

**Background:**

Molecular hydrogen (H_2_) is an attractive future energy carrier to replace fossil fuels. Biologically and sustainably produced H_2_ could contribute significantly to the future energy mix. However, biological H_2_ production methods are faced with multiple barriers including substrate cost, low production rates, and low yields. The C1 compound formate is a promising substrate for biological H_2_ production, as it can be produced itself from various sources including electrochemical reduction of CO_2_ or from synthesis gas. Many microbes that can produce H_2_ from formate have been isolated; however, in most cases H_2_ production rates cannot compete with other H_2_ production methods.

**Results:**

We established a formate-based H_2_ production method utilizing the acetogenic bacterium *Acetobacterium woodii*. This organism can use formate as sole energy and carbon source and possesses a novel enzyme complex, the hydrogen-dependent CO_2_ reductase that catalyzes oxidation of formate to H_2_ and CO_2_. Cell suspensions reached specific formate-dependent H_2_ production rates of 71 mmol g_protein_^−1^ h^−1^ (30.5 mmol g_CDW_^−1^ h^−1^) and maximum volumetric H_2_ evolution rates of 79 mmol L^−1^ h^−1^. Using growing cells in a two-step closed batch fermentation, specific H_2_ production rates reached 66 mmol g_CDW_^−1^ h^−1^ with a volumetric H_2_ evolution rate of 7.9 mmol L^−1^ h^−1^. Acetate was the major side product that decreased the H_2_ yield. We demonstrate that inhibition of the energy metabolism by addition of a sodium ionophore is suitable to completely abolish acetate formation. Under these conditions, yields up to 1 mol H_2_ per mol formate were achieved. The same ionophore can be used in cultures utilizing formate as specific switch from a growing phase to a H_2_ production phase.

**Conclusions:**

*Acetobacterium woodii* reached one of the highest formate-dependent specific H_2_ productivity rates at ambient temperatures reported so far for an organism without genetic modification and converted the substrate exclusively to H_2_. This makes this organism a very promising candidate for sustainable H_2_ production and, because of the reversibility of the *A. woodii* enzyme, also a candidate for reversible H_2_ storage.

## Background

Fossil fuel limitation and increasing atmospheric CO_2_ concentrations necessitate alternative energy carriers. Molecular hydrogen (H_2_) is an attractive carbon-free alternative that can be converted to energy without CO_2_ emission. It can be used as energy carrier for mobile applications (i.e., fuel cell powered vehicles) or as an intermediate energy storage system to store excess electrical energy that is produced in peak times from renewable sources [1]. Currently, H_2_ is produced mainly from fossil fuels by steam reforming and thus unsustainable and environmentally harmful [2]. Hence, new H_2_ production methods are required.

Biologically produced H_2_ provides a promising alternative for a sustainable H_2_-based energy economy. H_2_ production by biological systems can generally be classified into four different mechanisms: direct and indirect biophotolysis, photofermentation, and dark fermentation [3]. From these processes, the latter mechanism has so far the highest H_2_ evolution rates (HER). However, the major drawback of dark fermentations, e.g., from glucose, is the low H_2_ yield per substrate consumed and the limitations of agricultural production of the substrate [4]. A recently considered alternative substrate is formic acid/formate that could be produced from electrochemical reduction of CO_2_ or from synthesis gas, a very flexible substrate that can derive as by-product from steel mills or from waste gasification [5–7]. Conversion of formate to H_2_ proceeds according to the reaction:

$${\text{HCOO}}^{ - } + {\text{H}}_{2} {\text{O }} \rightleftharpoons {\text{HCO}}_{3}^{ - } + {\text{H}}_{ 2} \quad \Delta G^{{0}^{\prime }} = +\,1.3\,{\text{kJ mol}}^{ - 1}.$$

Microbial formate oxidation is catalyzed by multiple enzyme systems. Organisms such as some enterobacteria use a membrane-bound formate-hydrogen lyase system composed of membrane-associated hydrogenase and formate dehydrogenase subunits [8, 9]. *Clostridiaceae* or archaea such as *Methanococcus* can produce H_2_ from formate by the action of separate cytoplasmic formate dehydrogenases and hydrogenases [10]. The observed HERs for these organisms are typically very low and do not reach the levels for H_2_ production from other feedstocks [4]. One exception is the recently characterized organism *Thermococcus onnurineus*. This organism requires 80 °C for growth and formate-dependent H_2_ formation reached HERs that outcompete other dark fermentations for the first time [11, 12]. H_2_ production in this organism depends on a membrane-bound enzyme complex of formate dehydrogenase, hydrogenase, and Na^+^/H^+^ antiporter subunits that couples H_2_ formation to formate oxidation as well as energy conservation [13, 14].

A new enzyme of the bacterial formate metabolism has been discovered recently in the strictly anaerobic bacterium *Acetobacterium woodii* [15]. The enzyme named hydrogen-dependent CO_2_ reductase (HDCR) was the first described soluble enzyme complex that reversibly catalyzes the reduction of CO_2_ to formate with H_2_ as electron donor. CO_2_ reduction is catalyzed at ambient conditions with rates far superior to chemical catalysis [15–17]. Therefore, it could not only be used for H_2_ production but, depending on the application, for H_2_ storage as well. In the form of formate, the explosive gas could be stored and handled much easier and with an increased volumetric energy density [18]. H_2_-dependent CO_2_ reduction to formate by the HDCR has also been shown to be very efficient in whole cell catalysis with *A. woodii* [15]. However, the reverse reaction has not been addressed in detail so far.

In the present report, we describe the first characterization of formate-based H_2_ production with an organism harboring an HDCR complex. The results show that *A. woodii* has H_2_ production rates from formate of 66 mmol H_2_ g_CDW_^−1^ h^−1^ at ambient temperatures that are among the highest reported so far for an organism without genetic modification. Therefore, *A. woodii* is an efficient catalyst for H_2_ production and, considering the reversibility of the whole cell system, a potent catalyst for reversible H_2_ storage. In addition, *A. woodii* can grow with formate as sole carbon and energy source making it possible to produce cell mass and H_2_ with the same substrate.

## Results

### H_2_ production with resting cells

**Fig. 1:**
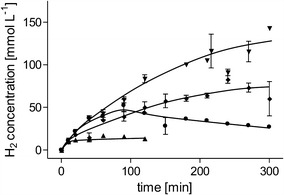
H_2_ production from formate by resting cells of *A. woodii*. Cells were grown with 20 mM fructose, harvested in the exponential growth phase, and suspended in buffer (50 mM imidazole, 20 mM KCl, 20 mM MgSO_4_, 4 mM DTE, pH 7) to a final protein concentration of 1 mg mL^−1^ (corresponding to a CDW of 2.3 g L^−1^) in anoxic serum bottles (gas phase 100% N_2_). The bottles were incubated in a shaking water bath at 30 °C. At the beginning of the experiment, sodium formate, potassium formate, ETH2120, NaCl, and ethanol (solvent of ETH2120 as negative control) were added as indicated. Triangles down, 300 mM sodium formate, 30 µM ETH2120 (dissolved in 100% ethanol), 20 mM NaCl; diamonds, 300 mM sodium formate, 20.5 mM ethanol, 20 mM NaCl; circles, 100 mM K-formate; triangles up, 100 mM sodium formate, 20.5 mM ethanol, 20 mM NaCl

The acetogenic bacterium *A. woodii* can utilize, among others, H_2_ + CO_2_, formate, or monosaccharides such as fructose as substrates for growth. In all three cases, acetate (or acetate + CO_2_ in the case of formate) is the major end product [19, 20]. Recently, we could show that the addition of the sodium ionophore ETH2120 (sodium ionophore III) led to a complete inhibition of acetate formation from H_2_ + CO_2_ and the two gases were completely converted to formate [15]. This opened the possibility to utilize *A. woodii* as catalyst for H_2_ storage. The hydrogen-dependent CO_2_ reduction activity could be addressed to a novel enzyme complex of a formate dehydrogenase and hydrogenase, named HDCR. Experiments with the purified enzyme showed that the catalyzed reaction proceeds with almost the same rate in the reverse reaction as well, making *A. woodii* a potential candidate for formate-based H_2_ production [15]. In this study, we analyzed this potential using whole cells of *A. woodii*. First, we grew the organism with fructose, a substrate to reach high cell densities relatively quickly (doubling time *t*_D_ = 4.7 h compared to 11 h with formate as substrate), harvested the cells, and incubated them in reaction buffer at a protein concentration of 1 mg mL^−1^ (corresponding to 2.3 mg_CDW_ mL^−1^). After addition of sodium formate to a final concentration of 300 mM, the cells produced H_2_ with an initial specific H_2_ productivity (qH_2_) of 52.2 ± 3 mmol g_protein_^−1^ h^−1^ (22.5 mmol g_CDW_^−1^ h^−1^) (Fig. 1). 0.6 mmol H_2_ was produced from 2.14 mmol formate consumed leading to a yield of H_2_ consumed per substrate consumed $\left( {Y_{{\left( {{\text{H}}_{2} /{\text{formate}}} \right)}} } \right)$ of 0.28 mol mol^−1^. It was surprising to observe these high H_2_ production rates since H_2_ is typically no major product from cells growing on formate; however, $Y_{{\left( {{{{\text{H}}_{2} } \mathord{\left/ {\vphantom {{{\text{H}}_{2} } {\text{formate}}}} \right. \kern-0pt} {\text{formate}}}} \right)}}$ was significantly decreased by the high amount of 0.45 mmol acetate produced alongside H_2_. The produced acetate results from the assimilation of CO_2_ or formate via the Wood–Ljungdahl pathway for autotrophic CO_2_ fixation of *A. woodii* [20, 21]. As shown recently for the reverse reaction of formate formation from H_2_ + CO_2_, we tried to decrease acetate formation by addition of the sodium ionophore ETH2120. Acetate formation in *A. woodii* is coupled to a sodium ion gradient for energy conservation across the cytoplasmic membrane that can be specifically diminished by the sodium ionophore. In the presence of 30 µM ETH2120, the final amount of H_2_ produced increased to 1.15 mmol from 1.68 mmol formate consumed. At the same time, acetate formation decreased to a final amount of 0.17 mmol acetate. In summary, addition of ETH2120 increased $Y_{{\left( {{{{\text{H}}_{2} } \mathord{\left/ {\vphantom {{{\text{H}}_{2} } {\text{formate}}}} \right. \kern-0pt} {\text{formate}}}} \right)}}$ to 0.68 mol mol^−1^. An alternative approach to the inhibition of acetate formation by ETH2120 is the depletion of the cells for sodium ions. In the CO_2_ reduction direction, sodium ion depletion showed the same effect on formate formation as ETH2120 but comes with much less cost for the fermentation. To test this for H_2_ production, we added potassium formate instead of sodium formate. Initial qH_2_ was identical to ETH2120 inhibited cells and the amount of H_2_ produced was more than double compared to the control (Fig. 1). However, after 100 min we observed reassimilation of H_2_ which decreased the product significantly. We interpret this result as an incomplete inhibition of sodium-dependent acetate formation due to sodium ion contamination in the potassium formate, which is 0.5% in ≥ 99.0% potassium formate used.

**Fig. 2:**
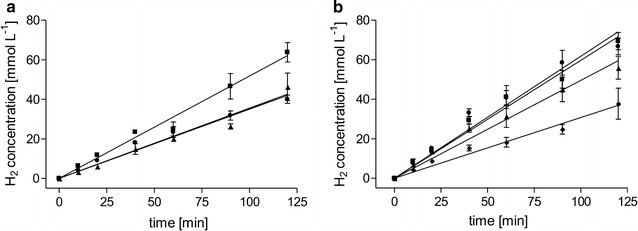
Influence of the growth substrate (**a**) and pH (**b**) on H_2_ production. **a** Cells were grown with 20 mM fructose (squares), 2 atm. H_2_ + CO_2_ (80:20 [v:v], triangles), or 100 mM sodium formate (circles). The experiment was performed as described for Fig. 1 using 300 mM sodium formate, 30 µM ETH2120, and 20 mM NaCl. **b** Fructose-grown cells were suspended in buffer (25 mM MES, 25 mM Tris, 25 mM MOPS, 25 mM CHES, 20 mM KCl, 20 mM MgSO_4_, 4 mM DTE, 20 mM NaCl) at pH 6 (circles), pH 7 (squares), pH 8 (triangles), pH 9 (diamonds). The experiment was started by the addition of sodium formate to a final concentration of 300 mM

**Fig. 3:**
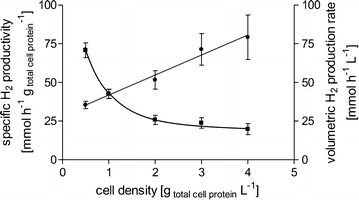
Influence of the cell density on volumetric and specific H_2_ production rates. Cells were grown with 20 mM fructose, harvested in the exponential growth phase, and suspended in buffer (50 mM imidazole, 20 mM KCl, 20 mM MgSO_4_, 30 µM ETH2120, 20 mM NaCl, 4 mM DTE, pH 7) to a final protein concentration of 0.5–4 mg mL^−1^ (corresponding to a CDW of 1.2–9.7 g L^−1^). Experiments were started by the addition of 100 mM sodium formate. Initial specific H_2_ production rates (squares) or initial volumetric H_2_ production rates (circles) are plotted against the cell density used

**Fig. 4:**
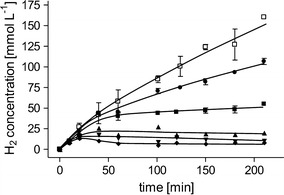
Influence of the formate concentration on H_2_ production. Cells were grown with 20 mM fructose, harvested in the exponential growth phase, and suspended in buffer (50 mM imidazole, 20 mM KCl, 20 mM MgSO_4_, 30 µM ETH2120, 20 mM NaCl, 4 mM DTE, pH 7) to a final protein concentration of 1 mg mL^−1^ (corresponding to a CDW of 2.3 g L^−1^). Experiments were started by the addition of 25 mM (diamonds), 50 mM (triangles down), 100 mM (triangles up), 200 mM (closed squares), 400 mM (circles), 600 mM formate (open squares)

In the initial experiments, we used fructose-grown cells as catalysts. An advantage of *A. woodii* is the wide range of possible growth substrates. Depending on the process and available substrate, cultivation of the cells on H_2_ + CO_2_ or directly on formate might be advantageous. qH_2_ in cells grown on H_2_ + CO_2_ was almost identical to formate-grown cells; however, with 21 mmol g_protein_^−1^ h^−1^ only 67% of the qH_2_ of fructose-grown cells was reached (Fig. 2a). pH dependency showed a decrease in qH_2_ with increasing pH within the tested pH range of 6–9 (Fig. 2b). Highest qH_2_ was observed at a pH of 6 with 37 mmol g_protein_^−1^ h^−1^. When using increasing cell densities, we observed a linear increase in HERs up to 79 mmol L^−1^ h^−1^ but a decrease in qH_2_ (Fig. 3). Maximum specific H_2_ production of 71 mmol g_protein_^−1^ h^−1^ (30.5 mmol g_CDW_^−1^ h^−1^) was observed at a protein concentration of 0.5 mg mL^−1^. At the same time, increasing cell densities led to higher accumulation of acetate and less production of H_2_, meaning that ETH2120 inhibition decreases at higher cell densities. In the next experiment, we tested inhibition of H_2_ production by increased formate concentrations. We tested formate concentrations from 25 to 600 mM. Within this range, initial H_2_ production rates did not change, with similar HERs up to 600 mM sodium formate tested, demonstrating that formate is not inhibiting the catalyst even at high concentrations. Final H_2_ concentrations increased with increasing initial formate concentrations (Fig. 4).

### H_2_ production in batch fermentation

**Fig. 5:**
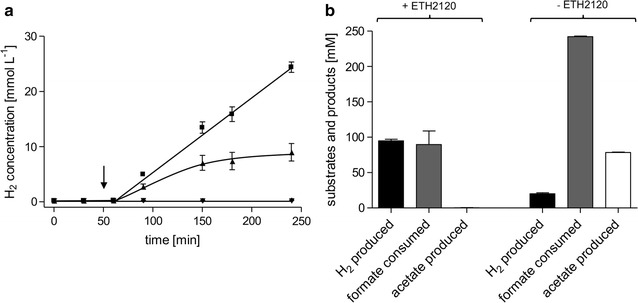
H_2_ production in closed batch fermentation by fructose-grown cells. *A. woodii* was grown in 50 mL carbonate-free growth medium with 20 mM fructose at 30 °C in a shaking water bath. At the point indicated, production phase was initiated by addition of sodium formate, ETH2120, or ethanol (solvent of ETH2120 as negative control). At this time point, the optical density of all cultures was between 0.35 and 0.45. H_2_ was measured in the gas phase and is plotted as mmol H_2_ per liter of growth medium (**a**). Substrate and product balance during the production phase (**b**) is shown as difference between *t* = 1 h (addition of formate and ionophore) and *t* = 24 h (end of fermentation). Squares, 300 mM sodium formate, 30 µM ETH2120; triangles up, 300 mM sodium formate, 20.5 mM ethanol; diamonds, 20.5 mM ethanol; triangles down, 30 µM ETH2120

The experiments described with resting cells showed that *A. woodii* is a promising catalyst for formate-dependent H_2_ production at ambient temperatures. For these experiments, cells were grown, harvested under anoxic conditions, and incubated in anoxic reaction buffer. This procedure is labor-intensive and requires sophisticated techniques to maintain anoxic conditions. To optimize this procedure, we wanted to abolish the medium exchange and establish H_2_ production directly in closed batch fermentation. Therefore, cells were grown with 20 mM fructose as substrate to mid-exponential growth phase (*t*_D_ = 4.7 h). At this point, formate was added with or without 30 µM ETH2120. Addition of the sodium ionophore led to an immediate growth arrest, whereas addition of formate alone had no effect on the growth rate (data not shown). After addition of formate, H_2_ was produced with a HER of 7.9 mmol L^−1^ h^−1^ and a qH_2_ of 65.9 mmol g_CDW_^−1^ h^−1^ (Fig. 5a). Without addition of ETH2120, the H_2_ evolution rate was 4.5 mmol L^−1^ h^−1^ initially, but decreased significantly after 1 h. After addition of formate, acetate was still produced alongside H_2_ when no ETH2120 was added (78.4 mmol L^−1^ after 23 h) (Fig. 5b). In contrast, cells in the presence of ETH2120 did produce acetate only in marginal amounts as side product (0.3 mmol L^−1^). $Y_{{\left( {{{{\text{H}}_{2} } \mathord{\left/ {\vphantom {{{\text{H}}_{2} } {\text{formate}}}} \right. \kern-0pt} {\text{formate}}}} \right)}}$ was 0.08 mol H_2_ mol formate^−1^ without and 1.06 mol mol^−1^ with ETH2120. The $Y_{{\left( {{{{\text{H}}_{2} } \mathord{\left/ {\vphantom {{{\text{H}}_{2} } {\text{formate}}}} \right. \kern-0pt} {\text{formate}}}} \right)}}$ above 1 can be explained by some H_2_ being produced from fructose still present in the fermentation (0.2 and 1.2 mmol L^−1^ of H_2_ where produced with and without ETH2120, respectively, from fructose alone). We observed that without addition of the ionophore, a total amount of 12.1 mmol formate was consumed from the initial 15 mmol (corresponding to a concentration of 300 mM). In the presence of ETH2120, this value decreased to 4.5 mmol. However, this can be explained from the different energetics of the reactions. Conversion of formate to acetate is highly exergonic, whereas conversion of formate to H_2_ is slightly endergonic, limiting the total conversion of formate in a batch system.

**Fig. 6:**
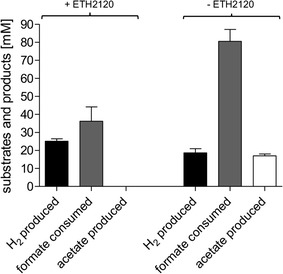
Substrate and product balance in closed batch fermentation with cells grown on formate. *A. woodii* was grown in 50 mL carbonate-free growth medium with 100 mM sodium formate at 30 °C in a shaking water bath to an optical density of 0.25–0.3. At this point, production phase was induced by adding 15 mmol sodium formate and 30 µM ETH2120 (+ETH2120) or 15 mmol sodium formate and 20.5 mM ethanol (−ETH2120). Substrate and product balance during the production phase is shown as difference between addition of formate and *t* = 24 h (end of fermentation)

Next, we wanted to further optimize the system by generating cell mass directly from formate as substrate, therefore testing a system independent on carbohydrates and using formate for growth and H_2_ production. Therefore, *A. woodii* was grown with 100 mM sodium formate (*t*_D_ = 11 h). These cultures already produced small amounts of H_2_ during growth (around 2 mmol L^−1^ before the switch to the production phase). To switch from growth to production phase, 15 mmol additional sodium formate (corresponding to 300 mM in the culture volume of 50 mL) with and without ETH2120 were added. As in the case for fructose-grown cells, H_2_ was produced immediately after addition of ETH2120 with a HER of 1.2 mmol L^−1^ h^−1^ and a specific production rate of 19 mmol g_CDW_^−1^ h^−1^. At the end of the fermentation, 25.1 mmol L^−1^ H_2_ was produced from 36.2 mmol L^−1^ formate consumed when ETH2120 was added ($Y_{{\left( {{{{\text{H}}_{2} } \mathord{\left/ {\vphantom {{{\text{H}}_{2} } {\text{formate}}}} \right. \kern-0pt} {\text{formate}}}} \right)}}$ = 0.69 mol H_2_ mol formate^−1^) (Fig. 6). Additional acetate was not produced after the addition of the ionophore. Without ETH2120, 18.6 mmol L^−1^ H_2_ and 17.0 mmol L^−1^ acetate were produced from 80.5 mmol L^−1^ formate. This results in a lower $Y_{{\left( {{{{\text{H}}_{2} } \mathord{\left/ {\vphantom {{{\text{H}}_{2} } {\text{formate}}}} \right. \kern-0pt} {\text{formate}}}} \right)}}$ of 0.23 mol H_2_ mol^−1^ formate. In comparison to fructose-grown cells, the final amount of H_2_ produced was much lower, even though the same amounts of formate were supplied. This could be an effect of the conditions established by the cells during the growth phase, e.g., growth on fructose leads to an acidification of the medium, whereas growth on formate increases the pH. Further studies need to address the optimal media composition depending on the substrate used for the growth phase. Nevertheless, the experiments with growing cells demonstrate in each case that the metabolism of *A. woodii* can be specifically switched from growth and acetate formation to H_2_ production by interfering with the sodium ion gradient across the membrane and thus dramatically increasing the yield coefficient $Y_{{\left( {{{{\text{H}}_{2} } \mathord{\left/ {\vphantom {{{\text{H}}_{2} } {\text{formate}}}} \right. \kern-0pt} {\text{formate}}}} \right)}}$.

## Discussion

**Fig. 7:**
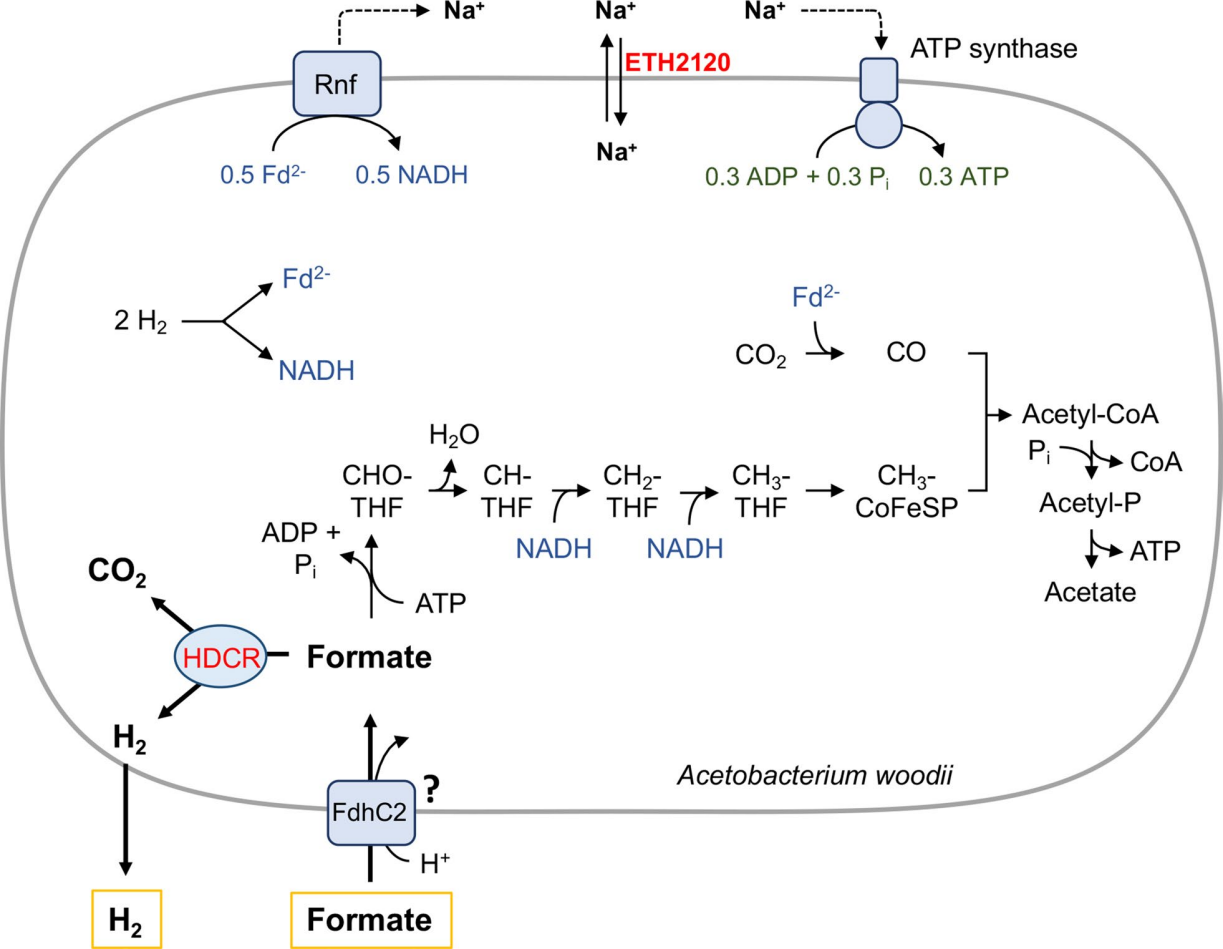
Model of formate-dependent H_2_ production with *A. woodii*. Formate can be used by *A. woodii* as carbon and energy source. Formate could be taken up by the putative formate transporter FdhC2. It is then bound to the cofactor tetrahydrofolate (THF) and reduced to a cofactor-bound methyl group. To generate the required reducing equivalents, part of the formate is oxidized to H_2_ + CO_2_ catalyzed by the HDCR. H_2_ is further oxidized by an electron bifurcating hydrogenase and CO_2_ is reduced to carbon monoxide (CO) which is fused to the methyl group resulting in the formation of acetyl-CoA and subsequently acetate. The Rnf complex generates a sodium ion gradient driven by the electron transfer from reduced ferredoxin to NAD^+^ that is then used by a sodium ion-dependent ATP synthase to generate ATP. The sodium ionophore ETH2120 collapses the membrane potential which inhibits ATP formation and could lead to ATP hydrolysis by the now uncoupled ATP synthase. This in turn inhibits conversion of formate to acetate because the first reaction is ATP dependent, resulting in sole conversion into H_2_ + CO_2_. CHO-THF, formyl-THF; CH-THF, methenyl-THF; CH_2_-THF, methylene-THF; CH_3_-THF, methyl-THF; CoFeSP, Corrinoid iron-sulfur protein; Fd, ferredoxin

In this study, we examined the H_2_ production capacity of the anaerobic bacterium *A. woodii*. This organism is a promising candidate for formate-based H_2_ production due to the recently identified reversible hydrogen-dependent CO_2_ reductase complex (HDCR), an enzyme able to reversibly reduce CO_2_ to formate with H_2_ as electron donor with so far exceptional catalysis rates. This enzyme catalyzes the first step in the Wood–Ljungdahl pathway, the pathway for CO_2_ fixation and energy conservation in this organism that has a wide substrate spectrum for growth ranging from monosaccharides, mono- and diols, H_2_ + CO_2_, and, especially important in this context, formate [20, 22]. However, without modification this organism produces mainly acetate as end product from most substrates [19]. As shown in this study, cells growing on formate produce only very little H_2_. Addition of high concentrations of formate to cells growing on formate or fructose led to immediate H_2_ production; however, H_2_ production rapidly slowed down and acetate was still produced. *A. woodii* can use H_2_ + CO_2_ for growth and acetate formation, and therefore this result is not unexpected since H_2_ + CO_2_ is the product of formate oxidation by the HDCR complex [15] (Fig. 7). The HDCR is not connected to the metabolism by electron carriers such as NAD^+^/NADH and it seems, from the results here, that it catalyzes formate oxidation unregulated if the formate concentration increases suddenly even if this provides no advantage for the cell. The independence of the HDCR from other metabolic processes makes it feasible to inhibit the major pathways for substrate conversion and growth by still retaining HDCR activity. As shown before, a very specific target for inhibiting the metabolism is the sodium ion gradient across the membrane that is built up during acetate formation and is necessary for energy conservation and growth. We assume that formate is imported by the putative formate transporter FdhC2 (Awo_c08050) whose gene is in close proximity to the HDCR gene cluster. FdhC2 could couple formate import to the proton gradient due to the similarity of the primary structure to the formate transporter FocA of *Escherichia coli* or *Salmonella typhimurium* [23, 24] (Fig. 7). In the next step, formate is reduced via the Wood–Ljungdahl pathway and the necessary reducing equivalents for this process are generated by oxidizing part of the formate via the HDCR. Addition of the ionophore should inhibit the reductive formate pathway without influencing the HDCR activity. This should stop acetate formation and result in the accumulation of hydrogen. At the same time, collapsing the membrane potential should be advantageous for uptake of the negatively charged formate molecule. As demonstrated in this study, neutralizing this gradient by adding a sodium ionophore (we used ETH2120) proved to be an effective switch from acetate to H_2_ production if formate is provided as substrate. It was possible to completely turn off acetate and biomass formation and reach yields $\left( {Y_{{\left( {{\text{H}}_{2} /{\text{formate}}} \right)}} } \right)$ of 100%. Comparing the total amount of formate consumed with and without ETH2120 showed that formate utilization stopped earlier when cells were inhibited by the ionophore. However, in this case formate was completely converted to H_2_ and this reaction is slightly endergonic ($\Delta G^{{0}^{\prime }} = +\,1.3\,{\text{kJ mol}}^{ - 1}$). The equilibrium constant of this reaction is therefore only 0.6. In the absence of the sodium ionophore, formate is mainly converted to acetate. This reaction is highly exergonic ($\Delta G^{{0}^{\prime }} = -\,110\,{\text{kJ mol}}^{ - 1}$ [25]) explaining the increased formate consumption. The thermodynamics of formate-based H_2_ production might seem as a disadvantage; however, the reaction close to the thermodynamic equilibrium allows simple adjustment of the direction of the reaction without additional energy supply. H_2_ can be produced from formate or stored in the form of formate without the input of much energy, a prerequisite for a reversible H_2_ storage material. Another very attractive property of formate-based H_2_ production is the complete conversion of the substrate to gaseous products. The substrate could be continuously supplied to the fermentation in the form of formic acid (at the same time providing a constant pH) resulting in the formation of H_2_ + CO_2_ only, circumventing any inhibition by dissolved products. Future studies need to address the long-term stability of the ionophore inhibited *A. woodii* system in such a continuous and pH-controlled fermentation. The price of the ionophore ETH2120 is a disadvantage considering the economic feasibility of the process. We used this compound to specifically study the effect of collapsing the membrane potential. However, with the gained knowledge that it is only necessary to inhibit the metabolism at any point it should be possible to identify other more inexpensive inhibitors. Alternatively, with the advent of genetic tools in acetogenic bacteria, mutations could be introduced to block key steps of the metabolism that stops acetate production and keeps the HDCR functional.

In summary, *A. woodii* and the corresponding enzyme HDCR turned out to be a very promising catalyst for formate-based H_2_ production and storage, as it operates at ambient temperatures with very similar reaction rates in the forward and reverse reaction. The specific H_2_ productivity (qH_2_) from formate observed with whole cells of *A. woodii* (66 mmol g_CDW_^−1^ h^−1^) is among the highest reported at ambient temperatures for an organism without genetic modification, highlighting the H_2_ production potential of this organism [4, 5]. Much higher qH_2_ are reported at 80 °C utilizing the thermophile *T. onnurineus* [12]. This organism uses a different enzyme system for formate-based H_2_ production, namely a membrane-bound enzyme complex consisting of a hydrogenase, formate dehydrogenase, and Na^+^/H^+^ antiporter subunits [13]. If *T. onnurineus* can also catalyze, the reverse reaction has not been shown so far. At ambient temperatures, the best results have been achieved using *E. coli* or other *Enterobacteria* such as *Citrobacter* in non-growing conditions [26]. Without genetic modification, *E. coli* has typically a low formate-dependent H_2_ productivity. However, by metabolic engineering including overexpression of the formate-hydrogen lyase enzyme, deletion of inhibitory pathways such as uptake hydrogenases and process optimization, the H_2_ productivity could be increased dramatically (144.2 mmol g^−1^ h^−1^ when products was removed continuously from the medium) [27, 28]. On the other hand, *E. coli* is inhibited by low concentrations of approximately 50 mM formate. This was addressed by using agar-embedded immobilized cells that were able to tolerate higher concentrations [29].

## Conclusions

This study demonstrated that *A. woodii* is an efficient H_2_ producer from the very flexible and inexpensive substrate formate. Together with our recent study on the reverse reaction, the results show that *A. woodii* can also be used as whole cell biocatalyst for the reversible storage of H_2_, by binding it to CO_2_ to produce formate and vice versa. Future studies need to address the process in a larger scale and in a continuous fermentation to analyze the stability and investigate alternatives to the expensive inhibitor ETH2120. Since any inhibition of the metabolism that does not affect the HDCR should be sufficient, other inhibitors or a genetic modification of the organism should be easy to find to improve the cost of the process.

## Methods

### Growth of *A. woodii*

*Acetobacterium woodii* (DSM 1030) was cultivated at 30 °C under anaerobic conditions. The defined carbonate buffered medium was prepared as described [30]. For closed batch fermentation, defined phosphate buffered medium was used and prepared as described [31]. Fructose (20 mM), formate (100 mM), or H_2_ + CO_2_ (80:20 [v/v]) was used as substrates. Growth was followed by measuring the optical density at 600 nm (OD_600_).

### Preparation of cell suspensions

The medium and all buffers were prepared using the anaerobic techniques described [32, 33]. All preparation steps were performed under strictly anaerobic conditions at room temperature in an anaerobic chamber (Coy Laboratory Products, Grass Lake, MI) filled with 95–98% N_2_ and 2–5% H_2_ as described [30]. *A. woodii* (DSM 1030) was grown in carbonate buffered medium till late exponential phase, harvested by centrifugation, and washed two times with imidazole buffer (50 mM imidazole–HCl, 20 mM MgSO_4_, 20 mM KCl, 4 mM DTE, 1 mg L^−1^ resazurin, pH 7.0). Cells were resuspended in imidazole buffer and transferred to Hungate tubes. The protein concentration of the cell suspension was determined as described previously [34]. To remove remaining H_2_ from the Hungate tube, the gas phase of the cell suspension was changed to N_2_ and the cells were stored on ice until use. For the experiments, the cells were suspended in the same buffer to a concentration of 1 mg mL^−1^ in 115-mL glass bottles. The bottles contained a final volume of 10 mL buffer under an N_2_ atmosphere and were incubated at 30 °C in a shaking water bath. Samples for substrate/product determination were taken with a syringe, cells were removed by centrifugation (15,000*g*, 2 min), and the supernatant was stored at − 20 °C until further analysis. For determination of H_2_, gas samples were taken with a gas tight syringe (Hamilton Bonaduz AG, Bonaduz, Switzerland) and analyzed by gas chromatography.

### Closed batch fermentations

*Acetobacterium woodii* (DSM 1030) was grown at 30 °C in 50 mL phosphate buffered medium in 115-mL glass bottles containing an initial gas phase of 100% N_2_. Samples for substrate/product determination were taken with a syringe and handled as described for the cell suspension experiments.

### Determination of hydrogen, formate, and acetate

For determination of H_2_, the gas samples were analyzed by gas chromatography on a Clarus 580 GC (Perkin Elmer, Waltham, USA) with a ShinCarbon ST 80/100 column (2 m × 0.53 mm, PerkinElmer, Waltham, MA, USA). The samples were injected at 100 °C with nitrogen as carrier gas with a head pressure of 400 kPa and a split flow of 30 mL min^−1^. The oven was kept at 40 °C and H_2_ was determined with a thermal conductivity detector at 100 °C. The peak areas were proportional to the concentration of H_2_ and calibrated with standard curves.

The concentration of formate was determined with an enzymatic assay using the formate dehydrogenase from *Candida boidinii* (Sigma-Aldrich, Munich, Germany). The assay contained in addition to the sample 1 U of enzyme in 50 mM potassium phosphate buffer (pH 7.5) and 2 mM NAD^+^. Formation of NADH was measured photometrically at 340 nm. Sodium formate was used for preparation of standard curves.

Acetate was measured using a commercially available enzymatic assay kit from R-Biopharm (Darmstadt, Germany).

### Chemicals

All chemicals were supplied by Sigma-Aldrich Chemie GmbH (Munich, Germany) and Carl Roth GmbH & Co KG (Karlsruhe, Germany). All gases were supplied by Praxair (Düsseldorf, Germany).
